# Mental Health and Psychosocial Support in Response to Onset of the COVID-19 Pandemic: Findings from Action Against Hunger’s Emotional and Stress Management Intervention in Ivory Coast, Liberia, and Sierra Leone

**DOI:** 10.1192/j.eurpsy.2023.1686

**Published:** 2023-07-19

**Authors:** K. Le Roch, A. Garriott, X. Phan, F. M. Bintu, S. P. Darciba, P. B. Koleti, S. M. Murray

**Affiliations:** 1 Mental Health and Psychosocial Support, Action contre la Faim, Paris, France; 2 Mental Health, Johns Hopkins Bloomberg School of Public Health, Baltimore, United States; 3 Mental Health and Psychosocial Support, Action Against Hunger, Freetown, Sierra Leone; 4 Action Against Hunger, Monrovia, Liberia; 5 Monitoring Evaluation Accountability and Learning, Action contre la Faim, Abidjan, Côte d’Ivoire

## Abstract

**Introduction:**

In 2020, as in the rest of the world, the COVID-19 pandemic spread in Africa and transformed people’s lives. Adding to the already existing burden of fragile health care systems, especially in low-resource settings, the pandemic response highlighted the need to address the health and well-being of populations in innovative ways. While research findings reported critical impacts on populations’ mental health, few studies assessed this progression within African countries. At the onset of the COVID-19 pandemic, Action Against Hunger (ACF), developed and delivered a brief Emotional and Stress Management Intervention (ESMI) to reduce symptoms of emotional distress and increase perceived social support through problem solving techniques and relaxation exercises among adults and youth living in vulnerable communities experiencing a relatively high prevalence of COVID-19 in urban and rural areas in Sierra Leone, Liberia and Ivory Coast.

**Objectives:**

The primary aim of this study is to evaluate whether individuals who received ESMI experienced changes in psychological distress and social support following the intervention and the association between change in psychological distress and change in social support for each country.

**Methods:**

This study consisted of secondary analysis of data collected via routine monitoring of activities by ACF for their ESMI programs implemented in community-based centers and health facilities from May to December 2020. Service delivery mechanisms were adapted to each context and setting (i.e., face to face vs. remote, health facilities vs. home visits, etc.). The main outcomes were psychological distress and social support measured with culturally relevant visual analogue scales. All analyses were performed separately for each country.

**Results:**

In total, 1,412 adults and youth (11-17 years old) benefitted from the intervention across all countries and 1,350 were assessed at follow-up. As a result, changes for psychological support and social support with mean scores difference at baseline and follow up were significantly different in all countries. Correlations between changes in distress and changes in social support varied by country, and ranged from negative in Liberia, (r = -.88, p = 0.001), to positive in Ivory Coast (r = .55, p = 0.001), and null in Sierra Leone (r = -.07, p = 0.11). Across countries, the most commonly reported presenting problems were fear of infection, stigma, and socio-economic difficulties, with coping strategies differing by country.

**Conclusions:**

At the onset of a pandemic crisis, low-intensity psychosocial support activities hold potential for reducing psychological distress and improving social support among adults and youth from vulnerable communities in the context of the COVID-19 pandemic.

**Disclosure of Interest:**

None Declared

